# Family caregivers' experiences of interaction with people with mild‐to‐moderate dementia in China: A qualitative study

**DOI:** 10.1111/ijn.12892

**Published:** 2020-09-23

**Authors:** Lili Yang, Huiling Ye, Qiuhua Sun

**Affiliations:** ^1^ School of Nursing Zhejiang Chinese Medical University Hangzhou China; ^2^ Department of Nursing Zhejiang University of Traditional Chinese Medicine First Affiliated Hospital Hangzhou China

**Keywords:** caregivers, dementia, family, interaction, nursing, qualitative

## Abstract

**Aim:**

This study aimed to explore the experiences of family caregivers interacting with people with dementia.

**Background:**

A majority of people with mild‐to‐moderate dementia live at home with family caregivers. This interaction creates positive experiences and challenges for these caregivers.

**Design:**

Descriptive phenomenological qualitative inquiry guided this study.

**Methods:**

This qualitative study involved semi‐structured interviews with the caregivers of people with mild‐to‐moderate dementia (*n* = 10). Data were collected from June to September 2018, and then data were thematically analysed.

**Results:**

Six categories of themes were identified from the interviews: (1) unexpected things often happen; (2) positive coping strategies; (3) sense of accomplishment because people with dementia actively participate in activities; (4) sense of frustration because of the reluctance of people with dementia to participate in activities; (5) hope for the happiness of people with dementia; and (6) want to have their own life.

**Conclusions:**

This study reveals that caregivers could positively interact with people with dementia through creating opportunities and arranging meaningful activities. Future research should focus on family management and training on how to help caregivers interact effectively with people with dementia.

## SUMMARY STATEMENT

What is already known about this topic?

- A majority of people with mild‐to‐moderate dementia live at home with family caregivers.
- Many caregivers suffer from depression, anxiety, fatigue, insomnia, family conflicts and low levels of life satisfaction.
- How caregivers interact with people with dementia at home remains unclear.

What this paper adds?

- Caregivers experience various feelings, such as a sense of accomplishment and occasionally frustration, when interacting with people with dementia.
- Caregivers stated that they wanted people with dementia to be happy and live their life. Thus, the caregivers changed their attitude, accepted the situation and organized meaningful activities for people with dementia.
- Caregivers employed various strategies for handling situations involving people with dementia and shared how they interacted with such individuals.

The implications of this paper:

- Care organizations that can offer certain services for people with dementia should be developed to lessen caregivers' burden.
- Community nurses should develop caregivers' strengths and enhance their abilities to improve interaction with people with dementia.
- Future research should focus on family management and training on how to help caregivers interact effectively with people with dementia.

## 1 INTRODUCTION

In 2018, over 50 million people with dementia (PwD) were reported worldwide, approximately 60% of whom are from developing countries, with the fastest growth in China (Prince et al., 2013; Winblad et al., 2016). Dementia is an incurable and progressive brain disease that typically causes cognitive impairment, communication difficulties and behavioural changes that often require lifelong assistance (WHO, 2016). At present, China has not established a long‐term care insurance system (Guo, 2017). The care for PwD is mainly provided at home; the caregivers are mainly from spouses, children, brothers and sisters in China (Dong, Guo, & Zhao, 2017). With an increasing aging population and an underdeveloped welfare infrastructure, Chinese family caregivers will encounter great challenges in rendering care for PwD (Sun, 2014). Thus, these conditions need the support of the family as a whole, and emphasis is placed on family harmony.

Many studies have shown that many caregivers suffer from depression, anxiety (Joling et al., 2015), fatigue, sleep disturbance (Liu et al., 2018; Villapando, 2015), family conflicts (Liddle et al., 2016) and low levels of life satisfaction. Hence, caregivers should find some strategies to alleviate their own physical and mental health. Previous qualitative studies examined the following values of caregivers and PwD: family and social relationships, safety and physical health, emotional well‐being, support for care needs and daily functioning, related services, medical care, support networks and meaningful activities (Heinrich, Uribe, Wübbeler, Hoffmann, & Roes, 2016; Macrae, 2010; Phinney, Chaudhury, & O'Connor, 2007). Furthermore, the positive and negative aspects of caregiving are of the same continuum—they could influence caregiver well‐being and satisfaction with life (Lethin et al., 2017). As the patient's cognitive function declines, communication and physical function decline; therefore, achieving positive social interactions becomes challenging (Mabire, Gay, Vrignaud, Garitte, & Dassen, 2016). In addition to accepting the situation, caregivers should adopt positive attitudes and create opportunities for PwD to engage in meaningful activities. Participating in meaningful activities positively influences the well‐being of PwD (Söderhamn, Landmark, Eriksen, & Söderhamn, 2013).

Interaction patterns, including preferred manner of expressing themselves and describing expectations on how each person behaves and reacts to one another, are established between caregivers and PwD through mutual support (Karner & Bobbitt‐Zeher, 2005; Lethin et al., 2017). Some meaningful interactions, such as housework (e.g., washing and cleaning), family games, handicrafts and outdoor activities (e.g., gardening and doing exercises), can be integrated into daily life. Caregivers can choose different forms of interaction on the basis of the interests and needs of PwD. For PwD, social relationships that involve positive social interactions are considered important factors that help enhance mood, cognition and quality of life (Dröes et al., 2016). A person‐centred caregiver is characterized by an element of a high‐quality relationship between caregivers and with PwD, and caregivers should develop harmonious relationships with PwD (McCormack, Karlsson, Dewing, & Lerdal, 2010).

In recent years, considerable research in Western countries focused on caregivers using positive family interaction strategies and participating in meaningful activities to influence the condition of PwD. Family caregivers in China spend a significant amount of time caring for PwD. Nonetheless, how caregivers interact with PwD at home remains unclear. Therefore, this qualitative study primarily aimed to describe the experiences of family caregivers who interact with PwD, and extraordinary skills are needed for family caregivers to manage the deteriorating cognitive function and consequent functional decline of PwD. This insight can provide a deep understanding of the needs of family caregivers, help PwD as well as their caregivers live meaningful lives and better inform health‐care professionals and nurses on caregivers' needs.

## 2 METHODS

### 2.1 Design

A descriptive phenomenological qualitative study was undertaken (Maxwell, 2013; Weaver & Olson, 2006). This approach lends to a deep understanding of the experience of caregivers who interact with PwD. An initial open‐ended question encouraged the caregivers to describe daily activities with PwD.

### 2.2 Participants

Caregivers of people with mild‐to‐moderate dementia were recruited from the Department of Neurology and Mental Health in Hangzhou in Zhejiang Province, China, and considered study participants in accordance with the following inclusion criteria: (1) age of 18 years and above; (2) main care for PwD (mild to moderate) of >3 months; and (3) at least 3 h of daily care for PwD.

Telephone contact was attempted for 23 eligible caregivers, and 10 caregivers completed the interviews. Eight women and two men from Hangzhou participated in this study. All were family caregivers for people with mild‐to‐moderate dementia. The duration of their experience with care services varied from 2 to 15 years. Seven family caregivers lived with the PwD in one flat; three lived separately from the PwD but within the same city. Data saturation being the guiding principle in sampling, the last interview analysed provided no new insights.

The characteristics of caregivers are displayed in Table 1.

**TABLE 1:** Characteristics of participants

Participant no.	Age of participant	Gender of participant	Relationship to PwD	Age of PwD	Gender of PwD	Living with the patient	Daily care time
1	64	Female	Daughter‐in‐law	98	Male	Y	12
2	69	Male	Husband	68	Female	Y	15
3	79	Female	Wife	86	Male	Y	13
4	77	Female	Wife	82	Male	Y	11
5	41	Female	Daughter	72	Female	N	5
6	74	Male	Husband	71	Female	Y	11
7	82	Female	Mother	65	Female	N	6
8	75	Female	Wife	80	Male	Y	8
9	55	Female	Daughter‐in‐law	82	Female	N	5
10	46	Female	Daughter	74	Female	Y	7

Abbreviation: PwD, people with dementia.

### 2.3 Data collection

An interview guide was developed and then refined via a discussion facilitated by the study team. The final guide included the following questions. How do you usually interact with PwD at home? What problems have you encountered during your interaction with the patient? What strategies do you use in response to the PwD's changed behaviours? What conditions do you want the PwD to keep in the future? What do you expect from your own life?

The interviewer explained the purpose of the interview and discussed the caregiver's right to discontinue at any time for any reason. Interview data were collected by audio recording. Interviews were conducted at the preferred time and place of the caregivers to provide a suitable environment for sharing their experiences. Once analyses were completed, a summary of the results was sent to the caregivers to request revisions.

Data were collected from June to September 2018 by an experienced nursing teacher who is a member of the research team. Interviewees who agreed to meet the researcher were contacted to arrange a convenient location and date. Eight interviews were conducted in participants' homes and two at the memory clinic. Interviews were conducted in a quiet room and lasted 30–45 min.

### 2.4 Data analysis

Interview recordings were transcribed, and the transcripts were read and analysed by the study team. A coding framework was developed through thematic analysis (Braun & Clarke, 2006). The study team became familiarized with the data by repeatedly listening to the interview recordings during transcription and repeatedly reading the transcripts. Memos were written to record the initial interpretation of each interview. The data were then stored and coded in NVivo9. In order to maintain qualitative rigour, two researchers independently coded the transcripts, compared and refined their coding categories and developed the themes. Initial codes were developed by collecting data with similar contents and subsequently converted into concepts and themes. Themes were generated and refined through an ongoing analysis. The process of refining the themes was thoroughly documented with the coding scheme developed before deciding on the best for the data.

### 2.5 Ethical considerations

Data collection was conducted after obtaining approval from the ethics committee institutional review board of the affiliated hospital of the university where the second author worked. Informed consent was obtained from each participant before the data collection. The participants voluntarily participated in this study and were informed of their rights to anonymity and confidentiality. The study was approved by the corresponding ethics committees of the institutions involved (The First Affiliated Hospital of Zhejiang Chinese Medical University, No. 2017‐KL‐071‐01). The participants were informed that the participation was voluntary and confidential and that they could withdraw from the study any time without explanation. At the start of the study process, they signed an informed consent form and agree to the recording. Recording materials are given strict confidentiality.

## 3 RESULTS

After the interview data were analysed, six core categories describing the family caregiver's experiences of interacting with PwD were identified. Table 2 shows the six themes.

**TABLE 2:** Main findings presented as themes

Themes
1. Unexpected things often happen
2. Positive coping strategies
3. Sense of accomplishment because PwD actively participate in activities
4. Sense of frustration because of the reluctance of PwD to participate in activities
5. Hope for the happiness of PwD
6. Want to have their own life

Abbreviation: PwD, people with dementia.

### 3.1 Theme 1: Unexpected things often happen

The caregivers experienced difficulty in anticipating possible problems or situations and establishing a strategy on how they and their family would handle PwD as their illness progressed. According to some caregivers, they had never been prepared for future occurrences, and this unpreparedness prevented them from creating appropriate plans.

She likes to do some handicrafts, and I am very supportive of her. However, she has stolen materials from stores for her handicrafts several times in the recent months. I'm worried about her when she goes out. Sometimes, I could not help but criticize and stop her. I do not know what to do. (Daughter, 5)

In addition, PwD often become angry or irritated for no obvious reasons, which can cause great trouble for caregivers.

She often suddenly gets angry. Then, she rushes out of the house and says something that we cannot understand. I wish I could make her happy, but her happiness is not in my hands anymore. I do not know when I will trigger her anger. (Husband, 6)

Continuous monitoring of PwD resulted in exhaustion, and some caregivers lacked sleep.

Recently, she woke up in the middle of the night and wanted to go to the convenience store. I wanted to accompany her and persuade her not to go. (Husband, 2)

### 3.2 Theme 2: Positive coping strategies

To manage some sudden or unexpected symptoms of PwD, caregivers not only accumulate experiences by consulting a doctor and searching for information in a network but also change their perceptions by positively adjusting their attitude. Avoiding possible conflict requires caregivers to help PwD apply the skills that still exist and adopt the concept of ‘we do things together’.

I learned how to handle sudden outbursts or unexpected situations. Now, I'm good at this, and I feel the situation is not so difficult anymore. When he becomes angry, I follow what he wants and let him do something that he is interested in to divert his attention. (Wife, 3)

Caregivers could consider and find ways to solve problems from a positive perspective. For example, they could focus on preserving their companionship with PwD. Several caregivers determined the behaviours and skills that PwD possessed. Some caregivers could retain the feeling of closeness to PwD. Caregivers could focus on the needs of PwD and promote their motivation to find meaning in their companionship.

The doctor told me that recalling our memories is good for him. We often look at our old photos, especially those about travel and family parties. He sometimes feels happy and talks about our past experiences. I no longer feel impatient. This is our happy time. (Wife, 8)

A majority of caregivers discussed the importance of accepting and accommodating cognitive deficits during an interaction with PwD. Some caregivers even attempted to learn the required skills from professionals or other individuals. Then, they could use methods to avoid conflict with PwD.

I am not afraid of letting others know. I told our neighbors about my mother's condition. Hence, they would contact me and help me when my mother stayed outside and had an unusual behavior. (Daughter, 5)

### 3.3 Theme 3: Sense of accomplishment because PwD actively participate in activities

Caregivers described the importance of establishing a good relationship with PwD by engaging in some activities. They emphasized that they made an effort to ensure that they were involved in activities inside and outside their homes. Many caregivers engaged in some activities, such as travelling, attending parties with acquaintances, participating in community activities and hanging out in the park, to relieve emotional problems, such as depression, worries and isolation, which they experienced with PwD.

I take her to the park every afternoon for one hour to listen to others singing. This activity has become a daily habit, and we enjoy this life. (Husband, 2)

Some caregivers intended to maintain activities in daily living with PwD. Thus, caregivers provided some tips that might promote comfort in familiar places and perform some activities at home. A few caregivers described the need to consider each person as an individual and to determine their personality, preferences and abilities.

I would make my mother do something that I think she can do, let her have something to do, and allow her to do things that she is willing to do. For example, I bought 50 pairs of socks and asked her to turn them over and repeat the procedure every day to exercise her fingers. (Daughter, 10)

Some caregivers stressed the importance of motivating the PwD to participate in some activities. They believed that engaging in the same activities had brought their families closer. Therefore, family served as a source of practical and emotional support for PwD.

Sometimes, we performed finger exercises with my father and my children. He was happy, and the family was close even if it was for only a short time. (Daughter‐in‐law, 1)

Some caregivers explained that most interactions with PwD occurred during their daily care activities. Some caregivers spoke of the importance of purposefully making additional opportunities for engage in activities.

He likes to participate in different activities with volunteers in our community entertainment center. He becomes more attentive, shows enthusiasm, and had much to talk about when he comes home. He is very happy. (Wife, 8)

### 3.4 Theme 4: Sense of frustration because of the reluctance of PwD to participate in activities

PwD exhibited psychological changes, such as apathy, lack of inhibition and anger, and these changes influenced the caregivers. In some instances, PwD became less socially active than before. Caregivers also experienced frustration and difficulty when interacting with PwD.

Sometimes, she became suddenly unhappy, stayed in her room, did not want to go out, and did not listen to my advice. It disrupted our original plan. (Husband, 6)

According to some caregivers, PwD were uninterested in some things that they used to do every day. In some cases, caregivers experienced difficulty in communicating with them.

He has been reading newspapers for two hours every day and discussing current affairs with me since he retired. Now, he seldom responds to me if I give comments on some things on the TV or in the newspaper that he is not interested in. (Wife, 4)

Some caregivers cried when they were talking about apathy of PwD. PwD were less interested in some activities in their daily lives, and this condition was also considered a burden.

She is at home all day, but she refuses to do housework and simply sits in a balcony in a daze. This situation will worsen, but we have no choice. (Mother, 7)

Common problems included apathy and reduced active participation in conversations, which in turn diminish the reciprocity of the relationship and lessen mutual enjoyment of each other's companionship.

I can discuss some things, but I have to start the conversation because she seldom carries on it. If I ask a question, she does not respond. (Daughter, 10)

We could visit others, but she wanted to go home again after half an hour. It was nice of someone to visit us. After an hour, she told our guests that they had to go. (Daughter, 5)

### 3.5 Theme 5: Hope for the happiness of PwD

Caregivers expected that PwD could become happier through their care. Some caregivers could derive meaning from choosing a positive attitude towards their unavoidable sufferings.

I hope he could be happy and his temper would be minimized. I want to make him feel comfortable, although I sometimes experiences some difficulties. (Daughter‐in‐law, 1)

Some caregivers described how they should change their mindset and natural responses in given situations to continue function as caregivers and simultaneously maintain some levels of emotional connection with PwD.

I have accepted her condition, and I have accumulated some experiences in caregiving when I interact with her. I know how to minimize my irritation toward her and keep her calm. I often share these experiences with others. (Daughter, 5)

Caregivers have prepared to overcome their challenges and attempted to change their attitude to maintain mental balance. Their goal was to create a different lifestyle with acceptable conditions.

I hope that she will have memories of good travel even if it would be tiring. I think that she will enjoy her time even for a moment. (Mother, 7)

Happiness for PwD was also expressed as moments of joy and comfort. They sometimes had fun in simple ways.

My mother likes to make bags and give them to our relatives. We would readily accept as long as she feel happy, although she creates more bags and making them seems tiring. (Daughter, 5)

Some of the caregivers described that provided PwD a greater sense of freedom and allowed them to be more focused on interacting with caregivers.

The goal I have is to keep my husband feel happy, so I want to live with him for as long as I live. (Wife, 3)

### 3.6 Theme 6: Want to have their own life

Some caregivers described how they need to find balance in their lives to be able to maintain relationships with other family members and friends. Caregivers had to stop previous hobbies and expressed a sense of loss when they had discontinued their previous activities. They also wanted to have some spare time to do things for themselves.

My husband and I have retired recently. We could have had enjoyed our retirement life. We could travel around if not for her. (Daughter‐in‐law, 9)

Caregivers also reported that their relaxed life has changed because of their caregiving responsibilities.

I experienced pressure from taking care of my father, but I think that this is what my life is now. Sometimes, I feel very tired. I want to relax with my friends on holidays, but who will take care of my father? (Daughter, 10)

For some caregivers, the idea of planning the future was too difficult. Some caregivers emphasized that they did not want to create future plans and they wanted to live one day at a time.

The trouble with dementia is that it could take either a long time or a short time. So, I do not know when the best time is to do it. (Husband, 6)

We tend not to think too far in advance. We would like to live each day as it comes. (Wife, 8)

## 4 DISCUSSION

This qualitative study focused on the experiences of caregivers interacting with PwD to enhance our understanding of their positive experiences and challenges. The psychological and behavioural problems of PwD mainly include suspicion, agitation, wandering behaviours and sleeping problems; thus, caregivers must remain alert and apply some strategies to cope with further challenges. In the beginning, caregivers were novice and had limited knowledge, but they acquired the required skills and became familiar with caring tasks over time. This study found that participating in meaningful activities positively affects the well‐being of caregivers and PwD. Six main themes emerged (see Table 2). Previous studies have reported that caregivers may actually find pride in providing care for PwD, because they seek to retain their former preferences (Janis & Maggie, 2016). Another previous study have mentioned that caregivers who are confident to take positive coping strategies are likely to handle effectively the symptoms of PwD and reduce the negative effects associated with caring (Papastavrou et al., 2011). Community nurses should assess the interaction between caregivers and PwD to develop comprehensive family management plans.Our study revealed that caregivers played important roles in interactions with PwD. Caregivers exhibit good manners and assist PwD in activities, although these caregivers experience some difficulties and express their intent to develop a relationship with PwD (Miriam, Amanda, Shirley, & Apam, 2016). Constituting a positive approach, meaningful activities and a remarkable relationship have been considered to benefit the PwD and the caregiver. Helping caregivers realize a special meaning in their interaction experiences is beneficial not only for PwD but also for caregivers themselves (Ablitt, Jones, & Muers, 2009; Van Beek & Gerritsen, 2010). To develop effective approaches for managing the needs of PwD, community nurses should support caregivers and consider relationship history, common lifestyles and interests. Caregivers should also develop adaptive skills to understand the causes of abnormal behaviours, meet the patients' reasonable needs and divert attention appropriately to cope with the challenges of interacting with PwD.

This study indicated that caregivers feel a sense of accomplishment when PwD actively participate and are frustrated when PwD are reluctant to participate in interactions; thus, caregivers organize activities for PwD. In the Chinese culture, the Chinese have learned to accept the fate of a given arrangement (Esther, Claudia, Fanny, & Pauline, 2007), and caregiving may be considered a duty that caregivers are obliged to perform (Holroyd, 2005). Many caregivers assume responsibility of taking care of PwD without assistance from other care systems and consequently face remarkable challenges (Sun, 2014). Family and friends play a crucial role because they have good insights into the behaviours, feelings and interaction needs of PwD. Activity coordinators can encourage communication and interaction through various activities and provide a useful communication link to ensure that individual needs, wishes and preferences are satisfied (Kolanowski, Van Haitsma, Meeks, & Litaker, 2014; Washington, Meadows, Elliott, & Koopman, 2011). Strategies such as accepting the situation, choosing a positive attitude and focusing on actively developing resources can help any caregiver. Community nurses can also train caregivers to engage in positive interactions with PwDs, which in turn will help them create a tolerant environment, formulate family interaction plans and expand social networks.

In addition to wanting PwD to be happy, caregivers likewise want them to live their life. Early and ongoing professional practical guidance and support are necessary to help caregivers acquire the required skills that allow them to effectively adapt to the changing behaviours of PwD; however, these interventions and support have been inadequate in China (Jane, Jiaying, Paul, & Philip, 2016). Comprehensive home care skill trainings may assist caregivers and PwD in modifying their day‐to‐day activities and behaviours to arrange meaningful activities. Therefore, community nurses should develop caregiver's strengths and enhance their skills to improve their interaction with PwD and address difficulties in caregiving. Day care organizations and short‐term care organizations should also be established to offer some professional services for PwD, reduce burden on caregivers and allow them to live meaningful lives.

### 4.1 Limitations

Several limitations should be noted. This study was conducted in Hangzhou. Family members living in this city might have a higher socioeconomic status than those living in less developed areas in China. The findings were based on one interview with each caregiver, but data collection was carried out in one geographical area in China. As such, this phenomenon may influence the generalizability of our findings. Future research should explore whether these interaction experiences differ from caregivers belonging to families with low socioeconomic status and exhibiting low education. We suggest that prospective quantitative studies should be developed to investigate the influential factors on caregivers interacting with PwD. In addition, we could help the caregiver to better interact with patients through intervention.

## 5 CONCLUSIONS

This study describes the experiences of caregivers in interacting with PwD, such as encountering various unexpected situations and feeling a sense of accomplishment and frustration. A central finding of our study states that caregivers could positively interact with PwD by employing positive coping strategies, creating opportunities and organizing meaningful activities. Such methods are essential for maintaining harmonious family relationships. Therefore, community nurses should use different strategies to improve their ability to interact with PwDs. Further studies can focus on the role of community nurses in improving the ability of caregivers to interact with patients and promoting further family management results.

## CONFLICT OF INTEREST

The authors have no conflicts of interest to disclose.

## AUTHORSHIP STATEMENT

Sun, Yang conceived the study and obtained research funding. Yang and Ye were responsible for data management and study design. Sun and Yang responsible for data analysis. All authors drafted and revised the manuscript.
